# Supplementary material for: Seroprevalence of antibodies against SARS-CoV-2 virus in Northern Serbia (Vojvodina): A four consecutive sentinel population-based survey study
Source: PLoS One. 2021 Jul 9;16(7):e0254516. doi: 10.1371/journal.pone.0254516 (PMC8270141; doi:10.1371/journal.pone.0254516)
Supplement: S1 Table — (DOCX) [file pone.0254516.s001.docx]

**S1 Table. Estimated number of SARS-CoV-2 seropositivity in population of Vojvodina, Serbia between April and September, 2020.**

| **Age group (years)** | **Point-of-care IgM/IgG test** | | | | | | | | | | | | **Line immunoassay test** | | |
| --- | --- | --- | --- | --- | --- | --- | --- | --- | --- | --- | --- | --- | --- | --- | --- |
|  | **End of April** | | | **End of May** | | | **End of June** | | | **End of September** | | | **End of September** | | |
|  | Total estimated anti-SARS-CoV-2  seropositives (n) | Minimum estimated anti-SARS-CoV-2  seropositives (n) | Maximum estimated anti-SARS-CoV-2  seropositives (n) | Total estimated anti-SARS-CoV-2  seropositives (n) | Minimum estimated anti-SARS-CoV-2  seropositives (n) | Maximum estimated anti-SARS-CoV-2  seropositives (n) | Total estimated anti-SARS-CoV-2  seropositives (n) | Minimum estimated anti-SARS-CoV-2  seropositives (n) | Maximum estimated anti-SARS-CoV-2  seropositives (n) | Total estimated anti-SARS-CoV-2  seropositives (n) | Minimum estimated anti-SARS-CoV-2  seropositives (n) | Maximum estimated anti-SARS-CoV-2  seropositives (n) | Total estimated anti-SARS-CoV-2  seropositives (n) | Minimum estimated anti-SARS-CoV-2  seropositives (n) | Maximum estimated anti-SARS-CoV-2  seropositives (n) |
| 0-4 | 0 | - | - | 0 | - | - | 0 | - | - | 9,174 | 1,934 | 24,267 | 7,027 | 852 | 22,998 |
| 5-14 | 3,190 | 377 | 11,287 | 5,323 | 642 | 18,516 | 5,643 | 698 | 19,592 | 24,518 | 12,098 | 42,637 | 23,914 | 12,268 | 40,636 |
| 15-29 | 6,431 | 1,744 | 16,240 | 12,317 | 4,542 | 26,268 | 23,107 | 11,699 | 40,292 | 60,893 | 41,855 | 84,218 | 77,170 | 56,460 | 101,367 |
| 30-64 | 33,035 | 21,049 | 49,114 | 45,411 | 30,404 | 64,900 | 67,531 | 48,432 | 91,114 | 157,281 | 128,729 | 189,244 | 168,975 | 140,617 | 200,450 |
| 65+ | 9,908 | 3,609 | 21,018 | 10,256 | 3798 | 21,904 | 17,694 | 8,578 | 31,780 | 29,691 | 17,884 | 45,613 | 42,479 | 28,963 | 59,351 |
| Total | 50,227 | 34,773 | 70,125 | 75,920 | 55,057 | 102,000 | 118,033 | 91,181 | 150,102 | 282,044 | 241,669 | 326,283 | 322,033 | 280,692 | 366,657 |
